# Subjective Nearness to Death and Emotional-Cognitive Complexity: An Integrative Model

**DOI:** 10.1093/geroni/igaf122.4203

**Published:** 2025-12-31

**Authors:** Sara Cohen-Fridel, Amit Shrira, Ehud Bodner, Yaacov Yablon

**Affiliations:** Bar-Ilan University, Ramat Gan, HaMerkaz, Israel; Bar-Ilan University, Ramat Gan, HaMerkaz, Israel; Bar-Ilan University, Ramat Gan, HaMerkaz, Israel; Bar-Ilan University, Ramat Gan, HaMerkaz, Israel

## Abstract

Lifespan developmental psychology emphasizes the role of emotional-cognitive complexity (i.e., the ability to hold conflicting emotions and thoughts) in late-life adjustment and functioning. The present study examines an integrative model that explores the relationship between subjective nearness to death (SNtD) and emotional-cognitive complexity, while considering the mediating role of the hostile world scenario (HWS). SNtD refers to how close one perceives oneself to death and is associated with negative psychological experiences. The HWS refers to an individual’s perception of actual or potential threats to their physical and psychological integrity. Partially supporting our hypotheses, our study (N = 1,073, ages 60-86) showed that some dimensions of the HWS mediated the relationship between SNtD and emotional-cognitive complexity. These findings highlight the theoretical contribution of the HWS in revealing the psychological mechanisms linking SNtD perception with emotional-cognitive adaptation in later life.

